# Growth charts: do they reflect healthy growth in Sri Lankan children?

**DOI:** 10.1186/s13104-016-2016-4

**Published:** 2016-04-09

**Authors:** V. P. Wickramasinghe, D. B. D. L. Samaranayake

**Affiliations:** Department of Paediatrics, University of Colombo, Colombo, Sri Lanka; Department of Community Medicine, University of Colombo, Colombo, Sri Lanka

**Keywords:** Growth charts, Sri Lankan children, Fat mass

## Abstract

**Background:**

Anthropometry has been used in the assessment of growth with “normal” range of growth being considered to span within ±2SD of median. Generally the practice is to make a child to grow within this standard deviation (SD) range. This study aims to investigate the hypothesis that all children with anthropometrical parameters within the designated “normal growth range” (within ±2SD of median), have a healthy body composition irrespective of the SD value.

**Results:**

Five to 15-year-old apparently healthy Sri Lankan children were studied. SD scores for height, weight and BMI, for age were calculated based on WHO 2007 standards. Sample was stratified according to age, sex, and SD score. %FM (assessed by isotope dilution methods using D_2_O) was compared between each SD group of each anthropometric parameter for gender and age. There were 278 (M/F:154/124) children. Non-parametric Kruskal–Wallis test revealed that the %FM of children differed significantly between SD groups even when they were within the accepted “normal limits of growth” and had higher levels of %FM in the upper SD groups.

**Conclusions:**

This study shows that Sri Lankan children have unacceptably high body fat at levels that have been designated by growth charts as “normal”. Children who are positioned at a higher SD score, +1 to +2SD, (within normal range) in the weight for age and BMI for age charts are mainly due to the deposition of fat rather than the growth in both fat and fat free compartments of body. Therefore the implication of using current cutoff values would be leading to the accumulation of excess fat in the body of those with higher SD values, but still considered to be within “normal” limits. Therefore in the absence of local standards, it would be important to revise the cutoff values of the WHO growth charts to suite Sri Lankan children which would be between −3SD and +1SD.

**Electronic supplementary material:**

The online version of this article (doi:10.1186/s13104-016-2016-4) contains supplementary material, which is available to authorized users.

## Background

Anthropometry has been an indispensable tool in the assessment of growth and nutrition in children. Height and weight had been the most frequently used anthropometric measures and body mass index (BMI) has been able to standardize the weight for height. Growth charts make a pictorial representation of the time line of growth and help to monitor the progress of growth more effectively and allow health professionals to take timely interventions. Cut-off values designated to be called “normal” were set principally based on statistical distribution of a growth parameter rather than on a biological endpoint having a cause and effect relationship. Considering the parameters’ had a Gaussian distribution, −2SD to +2SD of the median in the population distribution charts were taken as normal. Most of the charts have growth lines at 1 SD intervals on either side of the median for each age ranging from −3 to +3 SD. WHO growth charts for children above 5 years are developed using cross sectional data and from a limited number of geographical localities [1]. Therefore, their applicability across other ethnic groups based on same cutoff values is also questionable.

The usual practice in primary health care is to ensure that a child’s growth parameters lie within in this ‘normal range’. A child who is positioned below −2SD level, either in the weight for age or BMI for age chart is considered to be an undernourished. By treating him, one attempts to improve the nutritional status to a state above −2SD line. In order to achieve this, increased feeding takes place. Calorie dense, carbohydrate and fat rich diets, governed by the palatability of the child, are given. This may promote fat deposition in the body predisposing to cardiovascular and metabolic diseases in adulthood. Furthermore there is a group of children who are born with a birth weight lying below −2SD level and may not have the equal potential to grow. Any attempt to increase the weight beyond a certain point would result in addition of fat rather than having a healthy growth in both FM and FFM. Enthusiastically feeding these children aiming for rapid weight gain is not acceptable [2].

Higher fat content of body adversely affects health [3]. A child, who grows within the designated normal limits, should have a healthy body composition that would not increase the risk of developing non communicable diseases later in life. Therefore all children positioned within the designated normal range (−2SD to +2SD) in the commonly used growth assessment charts, height for age, weight for age and BMI for age, irrespective of their position within the ‘normal range’, should have a similar body composition, which should not predispose to adverse health outcomes later in life. In other words all children who have their growth parameters within ±2SD of the median, should have a healthy body composition with appropriate percentage fat mass (%FM) or fat mass to fat free mass ratio (FM:FFM). Therefore, within these limits, individuals should have a healthy body composition irrespective of the SD category. This study attempts to investigate the hypothesis that all children within the range of −2SD to +2SD (‘normal range’) from the median in growth charts (based on WHO 2007 growth standards) have a healthy body composition.

## Methods

Five to 15-year-old healthy Sri Lankan children were recruited from schools in Colombo. Schools were selected to represent children from different socioeconomic backgrounds and stratified according to age to get a fair representation of age and sex. Cluster sampling technique was adopted where one class from each grade, 1–10, were selected randomly. All students were invited to participate. Students to a class are allocated randomly therefore it was considered not necessary to select them randomly. Children who were ill during the preceding 2 weeks or those who were undergoing any special physical training that could have altered their body composition were excluded.

The study was conducted at the clinical laboratory of the Professorial Paediatric Unit of Lady Ridgeway Hospital for Children, Colombo, Sri Lanka from September 2004 to April 2005. Both parents and children were informed about the procedure. Informed written consent from parents and assent from children were obtained. The Ethical Review Committees of University of Colombo and Lady Ridgeway Hospital for Children approved the study.

The height was measured using a stadiometer (Surgical and Medical products, Australia) to the last completed 0.1 cm and weight was measured with minimal lightweight clothing to the closest 100 g, using an electronic weighing scale (Soehnle®, Soehnle-Waagen GmbH & Co, Germany) following standard techniques. BMI was calculated by weight/height^2^ (kgm^−2^). The total body water (TBW) was measured by isotope dilution method (D_2_O) and FFM and FM was calculated. Methodology is described in detail elsewhere [4]. All measurements were done by the first author.

Study population was categorised according to gender and age (5 ≤ to < 10 years and 10 ≤ to < 15 years). Standard deviation (SD) scores for weight for age (for 5 ≤ to < 10 year old group) height for age and BMI for age (for both age groups) were calculated based on WHO 2007 growth standards using lmsGrowth programme version 2.69 [5]. Weight for age was calculated only for 5 ≤ to < 10 year old group as it (weight for age) is used only in that age group. Children in each gender and age group were stratified into SD groups of <^−^3, ^−^3–^−^2, ^−^2–^−^1, ^−^1–0, 0–^+^1, ^+^1–^+^2, ^+^2–^+^3 and >^+^3 of the respective parameter. The mean %FM for each SD category was calculated. The mean %FM of each SD category within each gender and age group were compared using Kruskal–Wallis non-parametric test with Dunn-Bonferroni post hoc method with adjustment for multiple comparisons. A %FM cutoff of ≥25 % in boys and ≥32 % in girls were considered to be associated with adverse health outcomes [6].

Analyses were done using NCSS 2000 (Hintze JL, Kayswille, Utah, USA) statistical computer packages.

## Results

There were 124 girls and 154 boys. The characteristics of the study population are shown in Additional file 1: Table S1 according to age and gender. Additional file 1: Table S2 shows the median %FM for each weight for age SD category of the 5 ≤ to < 10 year group. The %FM kept on increasing with increase in weight and those in the SD categories above the median had unfavourably high fat content in the body. When %FM was compared between each SD category within the designated normal range (±2SD of the median), they were not similar always. That is children of a higher SD category had a higher percentage fat than those in a lower SD category. This clearly shows that those children enjoyed a higher position in the weight for age chart, mainly due to gaining in fat rather than due to a proportionate growth in both FM and FFM.

Additional file 1: Table S3 shows the analysis of the median %FM for each height for age SD category for both age groups. Within the designated normal limits (±2SD of the median), although an increase in the %FM is seen there was no statistically significant difference in the %FM in each SD categories. This denotes that when a child gains height, they mainly do it by increase in the FFM component and the contribution by the FM is not excess as seen in those who have a higher weight for age. However once again those in the higher SD groups have very high fat content of the body.

Additional file 1: Table S4 shows the analysis of median %FM for each BMI for age SD category in each age group. In the 5 ≤ to < 10 year aged boys the %FM in −2 to −1 SD category differed significantly from 0 to +1 and +1 to +2SD groups. In 10 ≤ to < 15 year aged boys, the %FM of SD categories below the median differed significantly with the ones above the median within −2SD to +2SD limits. In girls of both age groups, the median percentage fat mass increased in the higher BMI SD categories within the −2SD to +2SD limits, although this difference was not statistically significant.

Additional file 1: Table S5 shows the number of children who had a high percentage fat mass that is associated with adverse health outcomes. The number of children with adversely high fat percentage increased with the increase in the SD category.

## Discussion

Improvement in socioeconomic conditions and environment has improved the nutritional status (at least gain in weight) of children. However, this may have occurred due to excess deposition of fat rather than through a healthy growth of the body by having a proportionate growth in both FM and FFM thus keeping %FM in the body within normal limits. This is clearly shown by the fact that mean heights are more similar between SD groups than are weight and BMI in growth charts. Height represents physical growth of the body but weight represents growth of body as well as storage of excess energy ingested by way of macronutrients. BMI appears to have rectified the problem to some extent but not fully and this mainly due to increase in body weight in a population with relative short stature.

The disagreements in the body composition had mainly occurred between −2 and −1SD category and +1 to +2SD category. Therefore if the “normal range” is reduced arbitrarily by 1SD (i.e., −3SD to +1SD) these disagreements become minimal. Thus does this express the normal range of 2007 WHO standards are not suitable for Sri Lankan children? Therefore should ethnic specific charts be prepared which would have a different SD value distribution? In a previous study we have shown that current BMI cutoff values were not sensitive enough to detect high levels of percentage fat in Sri Lankan children irrespective of whether they live in Australia or in Sri Lanka [7, 8]. Furthermore even within ‘normal limits’ there are significant number of children with unfavourably high fat levels and the numbers are higher in high SD categories.

This study shows that although we have been using growth charts for many years, the cutoff values they represent do not provide the required information for effective monitoring of growth in children. Furthermore, there is a danger in taking action purely based on these growth charts and their cutoff values without taking body composition into account. Children who are considered to be within “normal” limits of growth, based on anthropometry, are actually having significant discrepancies in their body composition when compared across different SD categories. Therefore currently used growth charts raise some questions, such as, whether children growing within the designated “normal” range are truly having a healthy body composition. Can we always state that a child with a higher SD score, despite being within the designated “normal” limits have a healthy body composition. Does improving the weight of an undernourished child through nutrition rehabilitation and placing in a higher SD score, make them truly healthy? Can growth charts be used universally? Does assessment of growth based on these charts contribute to the ever rising epidemic of obesity? If a child is placed below −2SD is he an unhealthy child?

These issues have become important especially in today’s context with the increase in the nutritional status as well as with the trend of many becoming overweight and obese. Many with a high body fat content would be missed as we have set a higher threshold of anthropometric cutoff values to detect them becoming overweight or obese. Furthermore in the management of undernutrition, upper limit one should reach is not described, thus encouraging further gain in weight without any restrictions.

Therefore, perhaps new ways of diagnosing malnutrition may need to be developed. If a child, whose weight for age is at a comparable position to its birth weight, is growing steadily and is healthy, but weight for age since birth had been below −2SD, is it fair to call he/she an undernourished child, just because our definition of under nutrition is <−2SD on cross sectional data? Therefore, this study tries to highlight how growth charts could be misleading in instances. Based on these cutoff values high undernutrition rates have been shown and blanket feeding programmes have been initiated without taking individuality into account.

Main concern that is raised, is of the risk of developing NCD’s in future, as shown by the accelerated growth hypothesis [9] theory which suggests when children with a lower birth weight gain excess weight later in life (still remaining within upper normal limits), they will develop multiple metabolic problems even at a very young age. Similar results were shown in the meta-analysis by Baird and co-workers [2]. If we are thinking of controlling NCD in future, we have to start from a very young age and should be equipped with proper growth monitoring tools.

This paper tries to highlight the “inefficiencies” in the current cutoff values used in nutritional screening. This is expected to shed some light on where our children (especially those with birth weights on a lower side compared to westerners) should grow at younger age to have a healthy adulthood.

To add to this the WHO [10] also showed that BMI cutoff values for Asians should be lower than for Caucasians. Acting on these today some South Asian populations use lower BMI cutoff values for adults. So this data also shows that similar phenomena to a great extent, where the anthropometric cutoff values to have a healthy body composition lie at a lower level than what has been used in the case of European or north American children.

Therefore, the arguments put forward by this paper highlight the importance of assessment of body composition rather than use of simple anthropometric assessment methods in day to day clinical practice. Furthermore it shows the importance of validating cutoff values before they are used on a population other than that they are derived from. This may be more important for an economy in transition facing the ever rising epidemic of obesity and related metabolic complications.

South Asian populations who have higher amounts of body fat for any given BMI [8], have to seriously think how growth charts should be used to differentiate normal growth from abnormal growth. Should they be used by merely looking at the parameters or interpreted in the light of other clinical facts as well, especially considering how the child had been growing in the past including birth weight and their development. Further research is important especially in South Asian populations who have a higher risk of developing obesity related metabolic complications and are facing a rapid socioeconomic transition which affects their lifestyle resulting in changes in their physique.

## Conclusions

Cutoff values of different anthropometric parameters are statistically derived based on population distribution of the parameter. However this may not be the most suitable for many populations. Therefore if a growth chart is used in a population which was not involved in the original preparation, it should be validated or appropriate charts need to be developed to suit such populations. The pattern of growth of Sri Lankan children appears to be similar to white Caucasians, on whom these WHO charts were developed. However, limits within which an acceptable (or ‘normal’) body composition exists vary between Sri Lankan children and Caucasian children.

This denotes that a single growth chart can be used across different populations, but the cutoff values that to be used need to be different and in the case of Sri Lankan children cut of values for anthropometric parameters could be between −3SD and +1SD in the new WHO growth charts.

## Additional file

10.1186/s13104-016-2016-4 Five results tables are included. **Table S1** showing the characteristics of the study population, **Table S2–S4** showing the distribution of median percentage fat mass according to Weight-for-age SDS categories, Height-for-age SDS categories and BMI-for-age SDS categories and **Table S5** showing the distribution of children with increased %FM according to BMI-for-age SDS categories are presented.

## Abbreviations

SD: standard deviation
BMI: body mass index
FM: fat mass
%FM: percentage fat mass
FFM: fat-free mass
WHO: World Health Organisation
NCD: non-communicable diseases
