# Supplementary material for: Growth charts: do they reflect healthy growth in Sri Lankan children?
Source: BMC Res Notes. 2016 Apr 9;9:208. doi: 10.1186/s13104-016-2016-4 (PMC4826722; doi:10.1186/s13104-016-2016-4)
Supplement: Supplementary file 1 — 10.1186/s13104-016-2016-4 Five results tables are included. Table S1 showing the characteristics of the study population, Table S2–S4 showing the distribution of median percentage fat mass according to Weight-for-age SDS categories, Height-for-age SDS categories and BMI-for-age SDS categories and Table S5 showing the distribution of children with increased %FM according to BMI-for-age SDS categories are presented. [file 13104_2016_2016_MOESM1_ESM.docx]

|  | 5 ≤ to <10 year old | | 10≤ to <15 year old | |
| --- | --- | --- | --- | --- |
|  | Male | Female | Male | Female |
| N | 86 | 55 | 68 | 69 |
| Age | 7.6±1.3 | 7.4±1.3 | 12.2±1.6 | 12.2±1.2 |
| Height | 123.7± | 124.8±10.2 | 148.9±10.3 | 149.5±8.3 |
| Weight | 24.0±7.3 | 26.1±7.9 | 41.4±12.6 | 42.4±11.5 |
| BMI | 15.5±3.2 | 16.4±3.1 | 18.4±4.1 | 18.8±4.1 |
| Height Z score | -0.16±0.93 | 0.31±1.2 | -.03±0.93 | -0.30±0.9 |
| Weight Z score | -0.41±1.6 | 0.26±1.6 | - |  |
| BMI Z score | -0.58±1.9 | 0.09±1.6 | -0.21±1.8 | -0.14±1.5 |
| FM | 6.1±3.9 | 10.2±5.2 | 12.6±7.6 | 15.7±7.2 |
| FFM | 17.9±4.5 | 15.8±4.2 | 28.8±7.9 | 26.7±7.2 |
| Percentage FM | 23.8±9.6 | 37.6±10.6 | 28.7±11.3 | 36.4±10.2 |
| FM:FFM | 0.33±0.18 | 0.58±0.03 | 0.44±0.24 | 0.61±0.3 |
|  | | | | |

**Table S1:** Characteristics of the study population categorized according to the age and gender.

**Table S2:** Median %FM and its relation to weight for age SDS categories in 5 ≤ to <10 year old children.

|  | Male | | Female | |
| --- | --- | --- | --- | --- |
| SDS Category | Median %FM | Differ from | Median %FM | Differ from |
| 1. < ^-^3 | 14.74 | - | - | - |
| 2 **^.^**3-^-^2 | 17.66 | 5,6,7 | 24.69 | 7 |
| 3^.^ **^-^**2-^-^1 | 17.63 | 5,6,7 | 32.41 | - |
| 4. ^-^1- 0 | 22.51 | - | 32.89 | - |
| 5. 0 -^+^1 | 28.18 | 2,3 | 40.76 | - |
| 6. 1-^+^2 | 33.68 | 2,3 | 41.81 | - |
| 7. 2-^+^3 | 36.86 | 2,3 | 42.73 | 2 |
| 8. ^+^3 | 31.66 | - | - | - |
| Note: highlighted area shows the conventionally identified ‘normal range’ of the parameter. | | | | |

**Table S3:** Median %FM and its relation to height for age SDS categories in 5 ≤ to <10 and 10≤ to <15 year old children.

|  | 5 ≤ to <10 year Category | | | | | 10≤ to <15 year old category | | | | | |
| --- | --- | --- | --- | --- | --- | --- | --- | --- | --- | --- | --- |
|  | Male | | Female | | | Male | | | | Female | |
| SDS Category | Median %FM | Differ from | Median %FM | Differ from | | Median %FM | Differ from | | | Median %FM | Differ from |
| 1. < ^-^3 | - | - | - | | - |  | | - | - | | - |
| 2 **^.^**3-^-^2 | 14.74 | 6 | - | | - | 12.17 | | - | - | | - |
| 3^.^ **^-^**2-^-^1 | 17.83 | - | 32.28 | | - | 28.05 | | - | 33.66 | | - |
| 4. ^-^1- 0 | 20.77 | - | 37.26 | | - | 28.71 | | - | 38.10 | | - |
| 5. 0 -^+^1 | 24.90 | - | 32.46 | | - | 27.90 | | - | 36.78 | | - |
| 6. 1-^+^2 | 24.95 | 2 | 43.12 | | - | 37.62 | | - | 37.59 | | - |
| 7. 2-^+^3 | - | - | 35.93 | | - | - | | - | - | | - |
| 8. ^+^3 | - | - | - | | - | - | | - | - | | - |
| Note: highlighted area shows the conventionally identified ‘normal range’ of the parameter. | | | | | | | | | | | |

**Table S4:** Median %FM and its relation to BMI for age SDS categories in 5 ≤ to <10 and 10≤ to <15 year old children

|  | 5 ≤ to <10 year Category | | | | | 10≤ to <15 year old category | | | | |
| --- | --- | --- | --- | --- | --- | --- | --- | --- | --- | --- |
|  | Male | | | Female | | Male | | | Female | |
| SDS Category | Median %FM | Differ from | | Median %FM | Differ from | Median %FM | Differ from | Median %FM | | Differ from |
| 1. < ^-^3 | 17.65 | | 6,7 | 24.69 | - | 13.89 | 5,6,7 | 45.04 | | - |
| 2 **^.^**3-^-^2 | 17.02 | | 5,6,7 | 32.53 | 7 | 18.08 | 5,6,7 | 27.87 | | - |
| 3^.^ **^-^**2-^-^1 | 17.78 | | 5,6,7 | 32.46 | 7 | 19.99 | 5,6,7 | 35.01 | | - |
| 4. ^-^1- 0 | 22.51 | | - | 31.88 | 7 | 27.90 | 6,7 | 32.32 | | - |
| 5. 0 -^+^1 | 29.46 | | 2,3 | 39.29 | - | 33.45 | 1,2,3 | 39.09 | | - |
| 6. 1-^+^2 | 32.81 | | 1,2,3 | 40.19 | - | 37.85 | 1,2,3,4 | 39.20 | | - |
| 7. 2-^+^3 | 36.52 | | 1,2,3 | 45.51 | 2,3,4 | 45.24 | 1,2,3,4 | 47.47 | | - |
| 8. ^+^3 | 40.13 | | - | - | - | 13.89 | 5,6,7 | - | | - |
| Note: highlighted area shows the conventionally identified ‘normal range’ of the parameter | | | | | | | | | | |

**Table S5:** Distribution of children with increased %FM in each BMI for age SDS category in 5 ≤ to <10 and 10≤ to <15 year age groups, by gender

|  | 5 ≤ to <10 year Category | | | | | 10≤ to <15 year old category | | | | |
| --- | --- | --- | --- | --- | --- | --- | --- | --- | --- | --- |
|  | Male  (>25%FM) | | | Female (>32%FM) | | Male  (>25%FM) | | | Female (>32%FM) | |
| SDS Category | N | % | | N | % | N | % | N | | % |
| 1. < ^-^3 | 1 | | 11.1% | 1 | 33.3% | 0 | 0% | 4 | | 100.0% |
| 2 **^-^**3-^-^2 | 2 | | 15.4% | 2 | 50.0% | 2 | 25.0% | 2 | | 33.3% |
| 3^.^ **^-^**2-^-^1 | 2 | | 8.0% | 8 | 66.7% | 4 | 30.8% | 8 | | 72.7% |
| 4. ^-^1- 0 | 3 | | 23.1% | 3 | 50.0% | 7 | 63.6% | 7 | | 50.0% |
| 5. 0 -^+^1 | 6 | | 60.0% | 14 | 87.5% | 9 | 81.8% | 14 | | 73.7% |
| 6. 1-^+^2 | 9 | | 69.2% | 5 | 62.5% | 14 | 100.0% | 9 | | 90.0% |
| 7. 2-^+^3 | 7 | | 87.5% | 6 | 100% | 6 | 75.0% | 4 | | 80.0% |
| 8. ^+^3 | 2 | | 66.7% | 1 | 100% | - | - | 1 | | 100.0% |
| Total | 62 | | 34.0% | 40 | 71.4% | 42 | 61.8% | 49 | | 70.0% |
| Note: the highlighted area show the ‘suggested normal range’ of the parameter for Sri Lankan children | | | | | | | | | | |
